# Theoretical model of donor–donor and donor–acceptor energy transfer on a nanosphere

**DOI:** 10.1038/s41598-024-69718-4

**Published:** 2024-08-15

**Authors:** Anna Synak, Leszek Kułak, Piotr Bojarski

**Affiliations:** 1 Faculty of Mathematics, Physics and Informatics, University of Gdańsk, Wita Stwosza 57, 80-308 Gdańsk, Poland; 2 Faculty of Technical Physics and Applied Mathematics, Gdańsk University of Technology, Gabriela Narutowicza 11/12, 80-233 Gdańsk, Poland

**Keywords:** Excitation energy transfer, Nanoparticles, Monte Carlo simulations, Nanoscience and technology, Physics

## Abstract

In this study, we introduce a novel advancement in the field of theoretical exploration. Specifically, we investigate the transfer and trapping of electronic excitations within a two-component disordered system confined to a finite volume. The implications of our research extend to energy transfer phenomena on spherical nanoparticles, characterized by randomly distributed donors and acceptors on their surface. Utilizing the three-body Padé approximant technique, previously employed in single-component systems, we apply it to address the challenge of trapping within our system. To validate the robustness of our model, we conduct Monte Carlo simulations on a donor–acceptor system positioned on a spherical nanoparticle. In particular, very good agreement between the model and Monte Carlo simulations has been found for donor fluorescence intensity decay.

## Introduction

The phenomenon of non-radiative excitation energy transfer and trapping (NEET) within disordered luminescent systems, encompassing both solid and fluid solutions with a random distribution of donors and acceptors, has garnered considerable attention in scientific literature^1–7^. NEET processes have undergone extensive analysis across various molecular systems, including pure and mixed molecular crystals, porous structures, and diverse biological species, such as photosynthetic systems^8–11^. It serves as a spectroscopic ruler at the nanoscale, facilitating distance determination and proximity analysis in peptides, albumins, and other biomolecules^12–14^. Moreover, NEET proves valuable in elucidating interactions between nanomedicines and the biological environment^5,15^, and finds widespread application in biosensing^16,17^. Additionally, NEET offers insights into aggregation-related phenomena, including monomer fluorescence quenching and aggregation-induced emission^18–20^.

Recent studies have extended the investigation of energy transfer to various nanostructures, such as nanolayers, aimed at estimating local fluorophore concentration in porous, thin films and enhancing light emission or energy transfer efficiency^18,21–24^. In the original Förster model^1^, excitation energy transfers directly from an excited donor (D) to an unexcited neighboring acceptor (A) via dipole–dipole interactions. However, under conditions of high donor concentration, this transfer may be preceded by multiple excitation jumps between donors, provided there is partial overlap between donor absorption and fluorescence spectra. This stochastic process, known as excitation energy migration, has primarily been studied in random infinite systems like liquid solutions and amorphous solids. However, many physical systems, such as polymer mixtures undergoing phase separation, photosynthetic antenna complexes, quantum dots, nanoparticles, or proteins labeled with multiple chromophores, cannot be adequately represented by a uniform density of chromophores distributed in an infinite volume^25–27^.

In qualitative terms, the impact of finite volume on energy transfer can be elucidated as follows: molecules located near the volume's edge possess fewer neighboring chromophores compared to those nearer to the center. Consequently, the time required for energy propagation away from the initially excited molecule, when averaged across all starting positions and chromophore configurations within a finite volume, will be prolonged compared to an infinite volume with an equivalent chromophore density.

Moreover, even in the absence of traps, energy transport cannot transition to a diffusive regime over long time scales. Instead, the mean square displacement of excitation energy must converge to a constant value as the excitation probability becomes uniform throughout the finite volume. These finite-volume effects are most pronounced when the system's dimensions approach the critical radius for energy transfer.

Consequently, the finite dimensions of the system significantly alter energy transfer properties, which should manifest in the time-dependent behavior of luminescence characteristics such as donor fluorescence intensity decay or emission anisotropy decay. Therefore, theoretical models traditionally applied to describe disordered infinite systems in terms of energy migration and transfer may not be universally applicable to nanostructures of finite volume, specific geometries, and limited fluorophore numbers.

In the our previous works^28,29^, we discussed energy transfer in spherical nanoparticles with a finite radius $R$, where molecules attached to the surface act as energy donors. In our new paper, we analyze spherical nanoparticles where both donors and acceptors are attached to the surface, creating a more complex system with additional physical processes. The development of a specialized theoretical model capable of elucidating these intricate processes would not only fill a critical gap in our understanding but also pave the way for the precise design of nanoparticles with enhanced functionalities. From advanced imaging techniques to targeted drug delivery systems, the implications of such a model span a wide range of cutting-edge applications, promising breakthroughs in both research and practical implementations.

In this investigation, we introduce a novel approach utilizing the three-body Padé approximant method applied to the Green function, developed by our team. This innovative technique allows us to explore trapping phenomena within disordered systems confined to a finite volume. The three-body Padé approximant technique, central to our methodology, enables a nuanced understanding of the interplay between multiple excitations in a confined space. When extended to two-component systems, this technique reveals new facets of excitation dynamics that were previously inaccessible. Our study focuses on a finite volume system comprising immobile donor and trap molecules connected to a spherical nanoparticle.Excitations within this setup can be transferred between donors and from donors to acceptors (traps), with the trapping process assumed to be irreversible.

Furthermore, our approach provides a framework for exploring the effects of different spatial distributions and interactions among donors and acceptors. By varying parameters within our model, we can simulate different environmental conditions and predict how these changes impact the efficiency of energy transfer. This predictive capability is particularly valuable for designing experiments and interpreting experimental data in real-world applications.

Our methodology demonstrates remarkable versatility and can be applied to various molecular interactions. In this analysis, we specifically focus on dipole–dipole interactions. Our main objective is to determine the system's Green function, which allows us to compute all trapping and transport properties within the system.

The incorporation of multistep energy migration among groups of donors, followed by non-radiative excitation energy transfer to acceptors, is crucial for experimental measurements. These measurements require a sufficiently large number of initially excited donors to ensure accurate measurement of the fluorescent signal, considering potential disturbances and noise. The effectiveness of our model will be evaluated through Monte Carlo simulations.

Our Monte Carlo simulations not only corroborate the theoretical predictions but also highlight the practical applicability of our model. By demonstrating excellent agreement with the observed donor fluorescence intensity decay, we establish the credibility of our approach and its potential as a reliable tool for studying excitation transfer in various systems.

## Theoretical model

**Figure 1:**
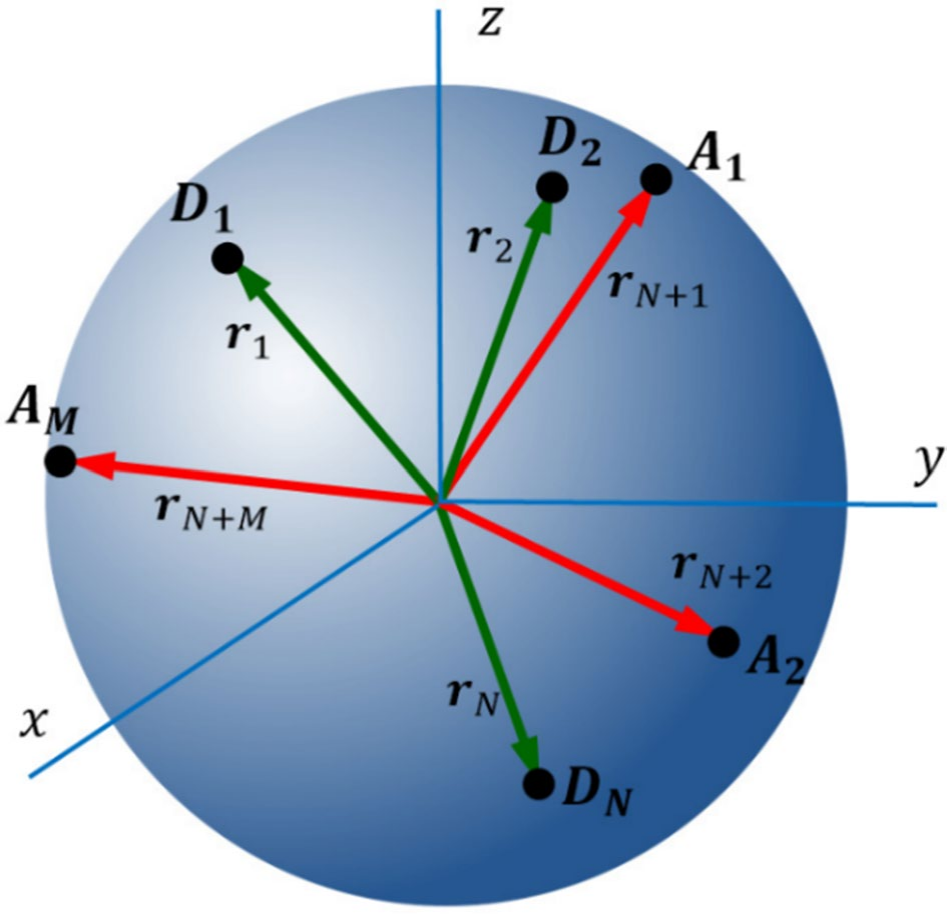
Geometrical relations between donors (D) and acceptors (A) on a spherical nanoparticle.

Let us consider a spherical nanoparticle with radius $R$ and surface $S=4\pi {R}^{2}$, containing $N$ donor molecules denoted as $D$ and $M$ excitation energy traps denoted as $A$ (acceptors), randomly distributed on the surface (refer to Fig. 1). The donor molecules are numbered from $1$ to $N$, and the trap molecules are numbered from $N+1$ to $N+M$. The individual configurations of molecules are described by the vector $\mathfrak{R}=\left({{\varvec{r}}}_{1},{{\varvec{r}}}_{2},\dots ,{{\varvec{r}}}_{N+M}\right)$.

Let us introduce the following notation for the transfer rate for excitation energy migration ${w}_{{x}_{i}{x}_{j}}^{DD}$ and ${w}_{{x}_{i}{x}_{j} }^{AA}$ between two donors $(D)$ and two acceptors $\left(A\right)$, respectively, while ${w}_{{x}_{i}{x}_{j}}^{DA}$ represents the energy transfer rate from a donor to an acceptor. Moreover, self-transfer does not occur, so ${w}_{{x}_{i}{x}_{i}}^{DD}\equiv 0$ and ${w}_{{x}_{i}{x}_{i}}^{AA}\equiv 0$. Each excited donor molecule, ${D}^{*}$, may undergo deactivation through fluorescence emission (with rate constant ${k}_{F}^{D}$ ), non-radiative transition (with rate constant ${k}_{q}^{D}$ ) or non-radiative energy transfer from ${D}^{*}$ to another donor molecule $D$ (with rate ${w}_{{x}_{i}{x}_{j}}^{DD}$). The transfer rates ${k}_{F}^{D}$ and ${k}_{q}^{D}$ are related to the characteristic time ${\tau }_{0D}$, by the relationship: ${\tau }_{0D}=1/\left({k}_{F}^{D}+{k}_{q}^{D}\right)$.

The dynamics of electronic excitation energy migration and transfer in the system of spherical nanoparticles are described by the following *master equation*

1 $$\frac{d{P}_{{x}_{i}{x}_{j}}\left(t\right)}{dt}=\sum_{k=1,k\ne i}^{N}{w}_{{x}_{i}{x}_{k}}^{DD}{ P}_{{x}_{k}{x}_{j}}\left(t\right)-\sum_{k=1,k\ne i}^{N}{w}_{{x}_{k}{x}_{i}}^{DD}{ P}_{{x}_{i}{x}_{j}}\left(t\right)-\sum_{k=N+1}^{N+M}{w}_{{x}_{k}{x}_{i}}^{DA}{ P}_{{x}_{i}{x}_{j}}\left(t\right) - \frac{{P}_{{x}_{i}{x}_{j}}\left(t\right)}{{\tau }_{0D}}, 1 \le i\le N$$

$$\frac{d{P}_{{x}_{i}{x}_{j}}\left(t\right)}{dt}=\sum_{k=1}^{N}{w}_{{x}_{i}{x}_{k}}^{DA}{ P}_{{x}_{k}{x}_{j}}\left(t\right)+\sum_{k=N+1,k\ne i}^{N+M}{w}_{{x}_{i}{x}_{k}}^{AA}{ P}_{{x}_{k}{x}_{j}}\left(t\right)-\sum_{k=N+1,k\ne i}^{N+M}{w}_{{x}_{k}{x}_{i}}^{AA}{ P}_{{x}_{i}{x}_{j}}\left(t\right)-\frac{{P}_{{x}_{i}{x}_{j}}(t)}{{\tau }_{0A}}, N+1 \le i\le N+M$$

where ${P}_{{x}_{i}{x}_{j}}(t)$ denotes the conditional probability density of finding the excitation at time $t$ on molecule ${x}_{i}$ given that at the initial moment $t=0,$ molecule ${x}_{j}$ was excited (where ${x}_{i}$ denotes all angular and spatial coordinates of the $i-th$ molecule). The initial condition is ${P}_{{x}_{i}{x}_{j}}\left(0\right)={\delta }_{ij} ,$ where ${\delta }_{ij}$ is the Kronecker delta.

The master equation can be expressed in matrix form as

2 $$\frac{d{\varvec{P}}\left(\mathfrak{R},t\right)}{dt}=\widetilde{{\varvec{W}}}\circ {\varvec{P}}\left(\mathfrak{R},t\right),$$

where

$${\widetilde{{\varvec{W}}}}_{jk}={w}_{{x}_{j}{x}_{k}}^{DD}-{\delta }_{jk}\left(\sum_{i=1}^{N}{w}_{{x}_{i}{x}_{k}}^{DD}+\sum_{i=N+1}^{N+M}{w}_{{x}_{i}{x}_{k}}^{DA}+\frac{1}{{\tau }_{0D}}\right), j\le N, k\le N$$

3 $${\widetilde{{\varvec{W}}}}_{jk}={w}_{{x}_{j}{x}_{k}}^{DA} , N+1\le j\le N+M, k\le N$$

$${\widetilde{{\varvec{W}}}}_{jk}=0, j\le N, N+1\le k\le N+M$$

$${\widetilde{{\varvec{W}}}}_{jk}={w}_{{x}_{j}{x}_{k}}^{AA}-{\delta }_{jk}\left(\sum_{i=N+1}^{N+M}{w}_{{x}_{i}{x}_{k}}^{AA}+\frac{1}{{\tau }_{0A}}\right), j\ge N+1, k\ge N+1$$

The solution to this equation is given by

4 $${\varvec{P}}\left(\mathfrak{R},t\right)=exp\left(t\widetilde{{\varvec{W}}}\right)\circ {\varvec{P}}\left(\mathfrak{R},t=0\right)$$

Information about the system is derived from the probability density, averaged over the spatial distribution $\mathfrak{R}$ of molecules, that the excitation of the molecule at time $t$ occurred at the point with the coordinate ${\text{r}}$

5 $$\mathcal{P}({\varvec{r}}\text{,}{\text{t}} \, \text{)=}{\langle \sum_{j=1}^{N+M}\delta ({{\varvec{r}}}_{j}-{\varvec{r}}){\left[{\varvec{P}}\left(\mathfrak{R},t\right)\right]}_{j}\rangle }_{\mathfrak{R}}\equiv \frac{1}{{\text{S}}^{\text{N}+\text{M}}}\int d{{\varvec{r}}}_{1}\dots \int d{{\varvec{r}}}_{\text{N+M}}\sum_{j=1}^{N+M}\delta ({{\varvec{r}}}_{j}-{\varvec{r}}){\left[{\varvec{P}}\left(\mathfrak{R},t\right)\right]}_{j}$$

where the bracket ${\langle \dots \rangle }_{\mathfrak{R}}$ signifies the ensemble average over the molecules’ distribution $\mathfrak{R}$.

The solution to the master equation is obtained using the Green's function method. The searched observable $\mathcal{P}({\varvec{r}} \, \text{, t })$ is then expressed in the following form

6 $$\mathcal{P}({\varvec{r}} \, \text{, t }\text{)=}\int d{\varvec{r}} \, \mathcal{G}({\varvec{r}},\,{\varvec{r}}{^{\prime}, }\text{t })\mathcal{P}({\varvec{r}}{\prime}\text{, 0)}.$$

Let us assume that the initial probability distribution ${\left[{\varvec{P}}\left(\mathfrak{R},t=0\right)\right]}_{j=1,N}$ depends solely on the spatial distribution of the excitation pulse, implying that only donor molecules were excited at the initial moment.

Then, the Green's function expressed by the elements of the matrix $\widetilde{{\varvec{W}}}$ assumes the following form

7 $$\mathcal{G}({\varvec{r}},\,{\varvec{r}}{^{\prime}, }\text{t })=\frac{\text{S}}{N}\sum_{j=1}^{N+M}\sum_{k=1}^{N}{\langle \delta ({{\varvec{r}}}_{\text{j}}-{\varvec{r}})exp{(t \widetilde{{\varvec{W}}})}_{jk}\delta ({{\varvec{r}}}_{\text{k}}-{\varvec{r}}{\prime})\rangle }_{\mathfrak{R}} .$$

The terms of the double sum above can be categorized into three classes: diagonal terms where $j = k$, and non-diagonal terms where $j\neq k$ for ${j\leq N}$ and $j > N$. Thus the Green function $\mathcal{G}({\varvec{r}},\,{\varvec{r}}{^{\prime}, }{\text{t}})$ can be represented as follows

8 $$\mathcal{G}({\varvec{r}},\,{\varvec{r}}{^{\prime}, }\text{t })={\mathcal{G}}^{SD}({\varvec{r}},\,{\varvec{r}}{^{\prime}, }\text{t }\text{) }\text{+}{\mathcal{G}}^{DD}({\varvec{r}},\,{\varvec{r}}{^{\prime}, }\text{t }\text{) }\text{+}{\mathcal{G}}^{DA}({\varvec{r}},\,{\varvec{r}}{^{\prime}, }\text{t })\text{,}$$

where

9 $${\mathcal{G}}^{SD}({\varvec{r}},\,{\varvec{r}}{^{\prime}, }\text{t })= \, \delta \left({\varvec{r}}-{\varvec{r}}{\prime}\right){\langle exp{\left(t \widetilde{{\varvec{W}}}\right)}_{11}\rangle }_{\mathfrak{R}},$$

10 $${\mathcal{G}}^{DD}({\varvec{r}},\,{\varvec{r}}{^{\prime}, }\text{t })= \, \left(N-1\right){\langle \delta \left({{\varvec{r}}}_{12}-{\varvec{r}}+{\varvec{r}}{\prime}\right) exp{(t \widetilde{{\varvec{W}}})}_{21}\rangle }_{\mathfrak{R}},$$

11 $${\mathcal{G}}^{DA}({\varvec{r}},\,{\varvec{r}}{^{\prime}, }\text{t })= \, \text{M }{\langle \delta \left({{\varvec{r}}}_{\text{1,N+1}}-{\varvec{r}}+{\varvec{r}}{\prime}\right) exp{(t \widetilde{{\varvec{W}}})}_{N+\text{1,1}}\rangle }_{\mathfrak{R}}.$$

The above Green functions can be interpreted as conditional probability densities of finding excitation at the point with the coordinate ${\varvec{r}}$ and the moment of time *t*, provided that for $t = 0$ the donor was excited at the point with the coordinate ${{\varvec{r}}}^{\boldsymbol{^{\prime}}}$.

In view of further considerations, it is convenient to perform the Fourier–Laplace transform of the Green function $\mathcal{G}({\varvec{r}},\,{\varvec{r}}{^{\prime}, }\text{t }).$ The transformed Green's functions are as follows:

12 $${\widehat{G}}^{SD}\left(\epsilon \right)={\langle {\left[{\left({\mathbb{E}}-{\varvec{W}}\right)}^{-1}\right]}_{11}\rangle }_{\mathfrak{R}} ,$$

13 $${\widehat{G}}^{DD}\left({\varvec{k}},\epsilon \right){=\left(N-1\right)\langle {exp\left(i{\varvec{k}}{{\varvec{r}}}_{12}\right)\left[{\left({\mathbb{E}}-{\varvec{W}}\right)}^{-1}\right]}_{21}\rangle }_{\mathfrak{R}} ,$$

14 $${\widehat{G}}^{DA}\left({\varvec{k}},\epsilon \right){=M\langle {exp\left(i{\varvec{k}}{{\varvec{r}}}_{1,N+1}\right)\left[{\left({\mathbb{E}}-{\varvec{W}}\right)}^{-1}\right]}_{N+\text{1,1}}\rangle }_{\mathfrak{R}} ,$$

where the matrix ${\mathbb{E}}$ has the following elements ${\mathbb{E}}_{jk}={\epsilon }_{D} {\delta }_{jk} , 1\le j\le N$**;** ${\mathbb{E}}_{jk}={\epsilon }_{A} {\delta }_{jk}, N<j\le N+M$ and ${\epsilon }_{A}=\epsilon +1/{\tau }_{0A }, {\epsilon }_{D}=\epsilon +1/{\tau }_{0D}$. The matrix ${\varvec{W}}={\left[{w}_{{x}_{i}{x}_{j}}^{XY}\right]}_{ i,j=1,\dots ,N+M}, X,Y\in \left\{A,D\right\}$ is called a transition matrix.

The Green's functions ${\widehat{G}}^{DD}\left({\varvec{k}},\epsilon \right),$ ${\widehat{G}}^{DA}\left({\varvec{k}},\epsilon \right)$ and ${\widehat{G}}^{SD}\left(\epsilon \right)$ are not independent. They collectively describe the probability density function, which should be normalized to unity

15 $${\epsilon }_{D}\left[{\widehat{G}}^{SD}\left(\epsilon \right)+{\widehat{G}}^{DD}\left({\varvec{k}}=0,\epsilon \right)\right]+{\epsilon }_{A}{\widehat{G}}^{DA}\left({\varvec{k}}=0,\epsilon \right)=1.$$

The decay of the donor fluorescence is obtained by inverting the Laplace transform of the function:

16 $$I\left(t\right)/{I}_{0}={\mathcal{L}}_{\epsilon }^{-1}\left({\widehat{G}}^{D}\left(\epsilon \right)\right)\equiv {\mathcal{L}}_{\epsilon }^{-1}\left({\widehat{G}}^{SD}\left(\epsilon \right)+{\widehat{G}}^{DD}\left({\varvec{k}}=0,\epsilon \right)\right).$$

## Expansion in a series with respect to fluorophores concentration

In the following, we develop an expression for ${\widehat{G}}^{D}\left({\varvec{k}},\epsilon \right)$, the Fourier–Laplace transform of ${\widehat{G}}^{D}\left(t\right),$ which describes the donor fluorescence decay (see Eq. (16)). First, we calculate the Green function ${\widehat{G}}^{DA}\left({\varvec{k}},\epsilon ,N,M\right)$, expanding it in a series in powers of the molecular surface density. For a system containing $N$ donors and $M$ acceptors distributed randomly over a sphere with a finite surface $S$, this expansion is as follows

17 $${\widehat{G}}^{DA}\left({\varvec{k}},\epsilon ,N,M\right)=\frac{M}{S}{\Lambda }_{1}^{DA}\left({\varvec{k}},\epsilon ,S\right)+\frac{\left(N-1\right)M}{{S}^{2}}{\Lambda }_{2}^{DA}\left({\varvec{k}},\epsilon ,S\right)+\frac{M\left(M-1\right)}{{S}^{2}}{\Lambda }_{3}^{DA}\left({\varvec{k}},\epsilon ,S\right) +\frac{M\left(M-1\right)\left(M-2\right)}{{S}^{3}}{\Lambda }_{4}^{DA}\left({\varvec{k}},\epsilon ,S\right)+\frac{\left(N-1\right)\left(N-2\right)M}{{S}^{3}}{\Lambda }_{5}^{DA}\left({\varvec{k}},\epsilon ,S\right)+\dots$$

The term ${\Lambda }_{1}^{DA}\left({\varvec{k}},\epsilon ,S\right)$ contains information about two-particle interactions (i.e. non-radiative excitation energy transfer). Similarly, ${\Lambda }_{2}^{DA}\left({\varvec{k}},\epsilon ,S\right)$ and ${\Lambda }_{3}^{DA}\left({\varvec{k}},\epsilon ,S\right)$ contain information about three-particle interactions, ${\Lambda }_{4}^{DA}\left({\varvec{k}},\epsilon ,S\right)$ and ${\Lambda }_{5}^{DA}\left({\varvec{k}},\epsilon ,S\right)$ four-particle interactions, and so on. If a general expression for the $n-th$ term is found, ${\widehat{G}}^{DA}\left({\varvec{k}},\epsilon ,N,M\right)$ could be calculated exactly. In practice, only the first three terms can usually be evaluated. For a random distribution of molecules on the finite surface of a spherical nanoparticle, we need to average the above expression over all possible molecular positions. The explicit form of functions ${\Lambda }_{i}^{DA}\left({\varvec{k}},\epsilon ,S\right)$ is presented in the Supplementary Material.

### Padé approximant for donor fluorescence decay

Padé approximants are commonly employed in statistical mechanics to approximate truncated power series expansions, such as Eq. (17). Regardless of the number of terms calculated in the expansion for ${\widehat{G}}^{DA}\left({\varvec{k}},\epsilon ,N,M\right),$ the result may behave poorly for large $N$ and $M$ or small $\epsilon$. In contrast, Padé approximants typically exhibit the correct asymptotic behavior in these limits and can serve as excellent approximations to ${\widehat{G}}^{DA}\left({\varvec{k}},\epsilon ,N,M\right).$

By introducing the following functions

18 $${f}_{2}\left(\epsilon \right)=\frac{1}{S}\int d{{\varvec{r}}}_{12}\frac{{w}_{{x}_{2}{x}_{1}}^{DA}}{{\epsilon }_{D}+{ w}_{{x}_{2}{x}_{1}}^{DA}} ,$$

19 $${f}_{3}\left(\epsilon \right)=\frac{1}{{S}^{2}}\int d{{\varvec{r}}}_{12}\int d{{\varvec{r}}}_{13}\left\{{\epsilon }_{A} C\left({{\varvec{r}}}_{12},{{\varvec{r}}}_{13},\epsilon \right)-\frac{{w}_{{x}_{3}{x}_{1}}^{DA}}{{\epsilon }_{D}+{ w}_{{x}_{3}{x}_{1}}^{DA}}\right\},$$

20 $${g}_{3}\left(\epsilon \right)=\frac{1}{{S}^{2}}\int d{{\varvec{r}}}_{12}\int d{{\varvec{r}}}_{13}\left\{{\epsilon }_{A} D\left({{\varvec{r}}}_{12},{{\varvec{r}}}_{13},\epsilon \right)-\frac{{w}_{{x}_{3}{x}_{1}}^{DA}}{{\epsilon }_{D}+{ w}_{{x}_{3}{x}_{1}}^{DA}}\right\},$$

where $C\left({{\varvec{r}}}_{12},{{\varvec{r}}}_{13},\epsilon \right)$ and $D\left({{\varvec{r}}}_{12},{{\varvec{r}}}_{13},\epsilon \right)$ are explicitly presented in the Supplementary Material, we obtain the following expression for the Fourier–Laplace transform of the donor fluorescence decay ${\widehat{G}}^{D}\left(\epsilon \right)$

21 $${\widehat{G}}^{D}\left(\epsilon \right)=\frac{1}{{\epsilon }_{D}}\left(1-M {f}_{2}\left(\epsilon \right)-\left(N-1\right)M{f}_{3}\left(\epsilon \right)- M\left(M-1\right) {g}_{3}\left(\epsilon \right)\right).$$

The Padé approximant to ${\widehat{G}}^{D}\left(\epsilon \right)$ is given by

22 $${\widehat{G}}^{D}\left(\epsilon \right)=\frac{\frac{1}{{\epsilon }_{D}}}{1+M {f}_{2}\left(\epsilon \right)+{\left(M {f}_{2}\left(\epsilon \right)\right)}^{2}+\left(N-1\right)M{f}_{3}\left(\epsilon \right)+ M\left(M-1\right) {g}_{3}\left(\epsilon \right)},$$

where ${{f}_{2}\left(\epsilon \right)>0, f}_{3}\left(\epsilon \right)>0,$ and ${g}_{3}\left(\epsilon \right)<0.$ Considering the sign of the ${g}_{3}\left(\epsilon \right)$ function (it is negative), it can be easily noticed, that including this term in the 3-body Padé approximant results in a slower fluorescence decay compared to the 2-body Padé approximant (see Eq. (26)). Conversely, inclusion of the ${f}_{3}\left(\epsilon \right)$ term associated with energy migration in the donor set, which results in energy transfer to the acceptor in the 3-body Padé approximant, accelerates the donor fluorescence decay. The final effect depends on the balance between both effects.

### 2-body approximation

By introducing the variables

23 $${\xi }_{A}=\frac{{R}_{0}^{DA}}{R}, {\xi }_{D}=\frac{{R}_{0}^{DD}}{R},$$

the function ${f}_{2}\left(\epsilon \right),$ representing interactions of two molecules (non-radiative energy transfer between one donor and one acceptor) is given by

24 $${f}_{2}\left(\epsilon \right)=\frac{a}{6 } \left\{ln\left|\frac{{\left(1+a\right)}^{2}}{1-a+{a}^{2}}\right|+2\sqrt{3}\left(arctg\left(\frac{2-a}{a\sqrt{3}}\right)+\frac{\pi }{6}\right)\right\} ,$$

where $a=\frac{1}{4}{\xi }_{A}^{2}{\left(\frac{1}{{\epsilon }_{D} {\tau }_{0D}}\right)}^{1/3}.$ By using the approximation described in the Supplementary Material (see Eq.(S21)), we obtain

25 $${f}_{2}\left(\epsilon \right)\approx \frac{\sqrt{3} \pi }{18} {\xi }_{A}^{2} {\left({\epsilon }_{D} {\tau }_{0D}\right)}^{-\frac{1}{3}} .$$

Thus, the 2-body Padé approximant of the Fourier–Laplace transform of the donor fluorescence decay is

26 $${\widehat{G}}^{D}\left(\epsilon \right)\approx \frac{\frac{1}{{\epsilon }_{D}}}{1+M {f}_{2}\left(\epsilon \right)+{\left(M {f}_{2}\left(\epsilon \right)\right)}^{2}}.$$

### 3-body approximation

The function ${f}_{3}\left(\epsilon \right),$ describing the interactions of three molecules (2 donors and 1 acceptor), when time is measured in units of ${\tau }_{0D}$ with excluded natural decay $exp\left(-\frac{t}{{\tau }_{0D}}\right),$ assumes the following form (see Supplementary Material)

27 $${f}_{3}\left(\epsilon \right)=\frac{1}{64 {\pi }^{2}} {\epsilon }^{-2/3} {\xi }_{A}^{2} {\xi }_{D}^{2}{ \mathcal{J}}_{3}\left(\alpha , \epsilon \right) ,$$

where the integral ${\mathcal{J}}_{3}\left(\alpha , \epsilon \right)$ can be approximated by

28 $${\mathcal{J}}_{3}\left(\alpha , \epsilon \right)=\frac{1}{\left(a+b {\alpha }^{2}\right)}, a=0.02712, b=0.04649, \alpha =\frac{{\xi }_{A}}{{\xi }_{D}}\equiv \frac{{R}_{0}^{DA}}{{R}_{0}^{DD}} .$$

Similarly, the function ${g}_{3}\left(\epsilon \right)$ also describing the interactions of three molecules (1 donor and 2 acceptors), can be calculated as

29 $${g}_{3}\left(\epsilon \right)=\frac{1}{16} {\epsilon }^{-2/3} {\xi }_{A}^{4} { \mathcal{K}}_{3}, { \mathcal{K}}_{3}=-0.82764 .$$

The expressions from Eq. (25), (27) and (29) inserted into Eq. (22) finally give us the studied 3-body Padé approximant for ${\widehat{G}}^{D}\left(\epsilon \right)$.

## Results and discussion

**Figure 2:**
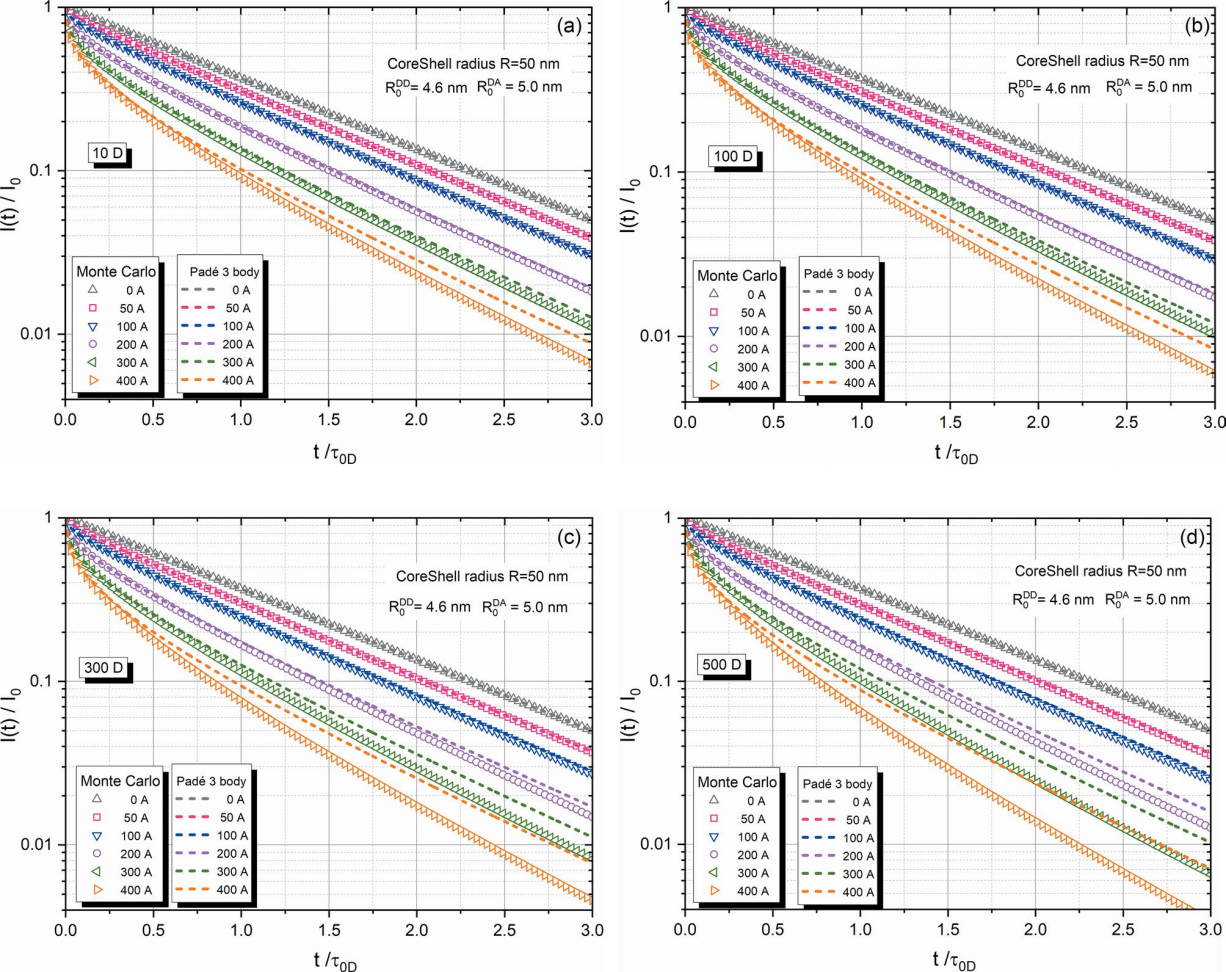
**a-d.** Donor fluorescence intensity decay in the presence of acceptors: (**a**) 10 donors, (**b**) 100 donors, (**c**) 300 donors and (**d**) 500 donors.

Figures 2a–d illustrate the results of donor fluorescence intensity decay in the presence of various numbers of acceptors. To provide a representative case for comparison, we selected a system comprising a nanoparticle with a radius $R= 50$ nm, labeled with donors and acceptors characterized by ${R}_{0}^{DD}=4.6$ nm and ${R}_{0}^{DA}=5$ nm^9^. The results in Figs. 2a–d show the decay for different numbers of donors: 10 donors in Fig. 2a, 100 donors in Fig. 2b, 300 donors in Fig. 2c and 500 donors in Fig. 2d. This systematic variation allows us to evaluate the influence of energy migration preceding energy transfer on the donor fluorescence decay. Geometric symbols in the figures represent the results obtained from Monte–Carlo simulations, while the scattered lines depict the theoretical predictions within the three-body Padé approximant. It is evident from all figures that in the absence of acceptors, the predicted donor fluorescence decay follows a single exponential character and can be described by the same curve regardless of the number of donors. A similar result can be observed for three-dimensional solutions. However, as the number of acceptors increases, the fluorescence intensity of donors decays significantly faster, exhibiting a non-exponential character due to effective excitation trapping. Comparing the curves obtained for the same number of acceptors but different number of donors reveals that the donor intensity decays faster for systems with stronger energy migration.

The agreement between the Monte-Carlo result and the theory within the three-body Padé approximant is excellent up to 200 acceptors for small and moderate number of donors (Fig. 2a, b). Even in the case of a large number of donors, corresponding to strong energy migration, the agreement remains very good up to 100 acceptors. These results are surprisingly good considering the nature of the three -body Padé approximant described earlier. However, for an extremely high number of acceptors, the Monte–Carlo results predict faster decays compared to the theoretical predictions, reflecting the limitation of the Padé approximant.

**Figure 3:**
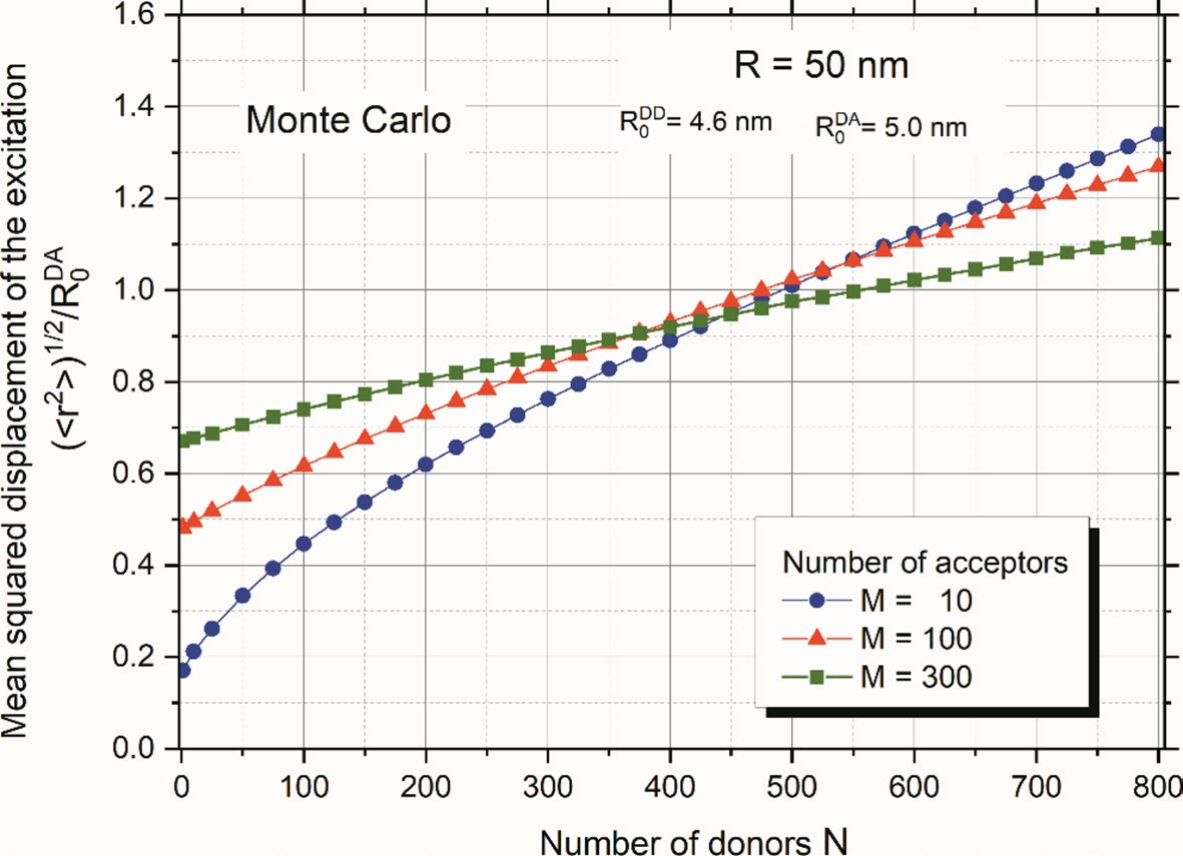
Monte Carlo results of relative mean squared excitation displacement as a function of the number of donors for three fixed numbers of acceptors.

During energy migration and transfer, excitation traverses many sites on a nanoparticle, making it crucial to understand the spatial extent of this stochastic process. A useful metric for this is the relative mean squared displacement of the excitation energy, denoted as ${(<{r}^{2}>)}^{1/2}{/R}_{0}^{DA}$. Figure 3 illustrates the Monte-Carlo results of the relative mean squared excitation displacement versus the number of donors for three fixed numbers of acceptors. It is evident that the range of excitation walk increases significantly with the number of molecules present on the nanoparticle, reaching values between 1 and 1.35 depending on the number of acceptors. These values are substantial, indicating that excitation may reach distant sites from the initially excited one. Hence, energy migration should not be regarded as a purely local process in terms of nanoparticle size.

Figure 3 illustrates that in the case of a single donor (Förster case), the curves start higher, corresponding to a greater number of acceptors. This occurs because the emission of photons by the initially excited donor outweighs the energy transfer to the acceptors. As the number of donors increases, the migration of energy within the set of donors becomes increasingly crucial in determining the relative mean squared excitation displacement value. When the number of donors significantly exceeds the number of acceptors, energy migration becomes dominant. In such scenario, the curves reverse, with the curve corresponding to the smallest number of acceptors being the highest. This effect directly results from increased energy trapping with a growing number of acceptors, assuming a fixed number of donors.

**Figure 4:**
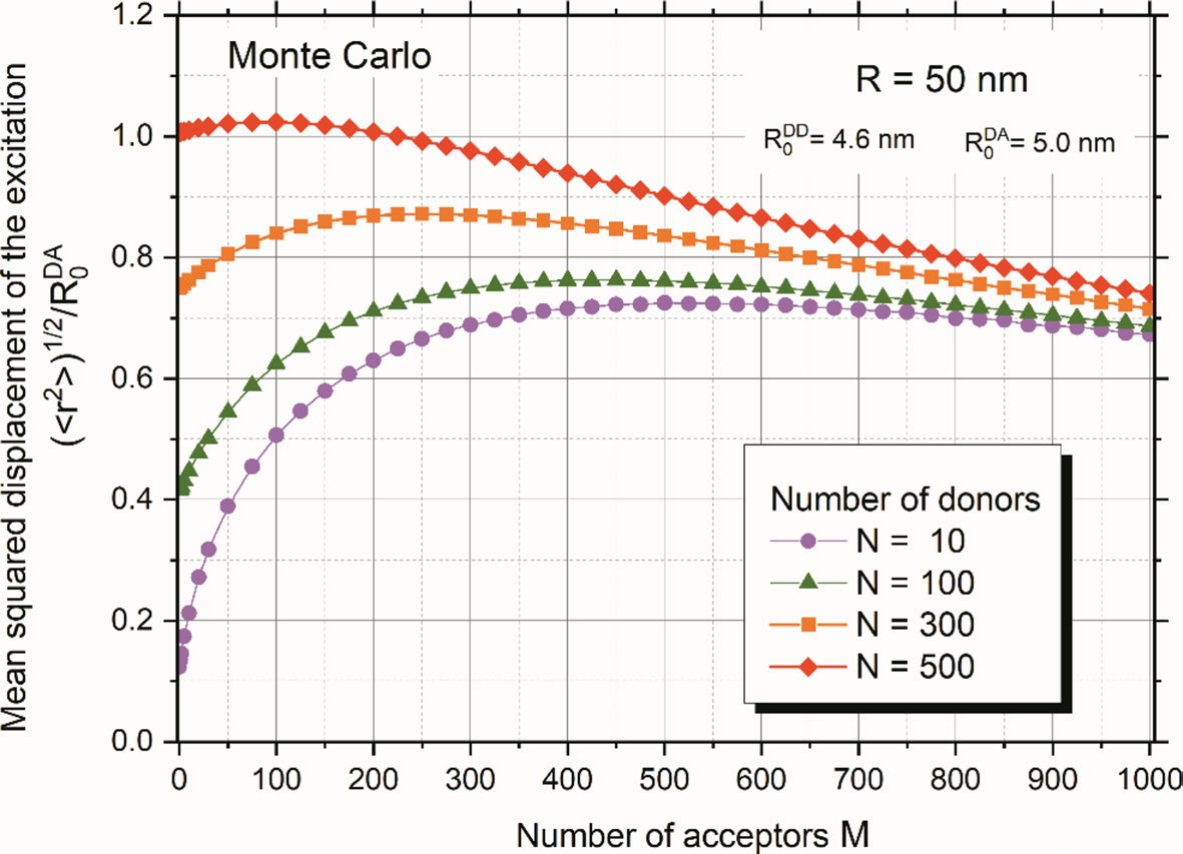
Monte Carlo results of relative mean squared excitation displacement as a function of the number of acceptors for four fixed amounts of donors: *N* = 10;100;300;500.

Figure 4 depicts the results of Monte Carlo simulations of relative mean squared excitation displacement as a function of the number of acceptors for four fixed numbers of donors**.** In the absence of acceptors ($M=0$), the curves start higher, corresponding to a greater number of donors. This is attributed to the increasing importance of energy migration within the set of donors when energy transfer to acceptors is absent, leading to a higher relative mean squared excitation displacement with more donors. As the number of acceptors increases (with a fixed number of donors), the relative mean squared excitation displacement decreases due to increasing energy trapping. When the number of acceptors significantly exceeds the number of donors, the influence of energy migration diminishes, and all four curves asymptotically approach the Förster case.

## Conclusion

The presented model introduces a novel perspective on energy migration and trapping on a spherical nanoparticle with chemically linked donors and acceptors. This model reveals that the dynamical behavior of the system is not only exquisitely responsive to the number of donors, acceptors, and the radius of the nanoparticle, but also showcases how these factors interplay in novel ways. Monte Carlo simulations, conducted for this innovative model, not only affirm the utility of the three-body Padé approximant but also highlight its novelty in capturing complex interactions.

Intriguingly, certain deviations observed between theoretical predictions and results of the Monte Carlo simulations at long times originate from the inherent characteristics of the Padé approximant, shedding new light on its limitations. While remarkably effective for scenarios involving a relatively modest number of attached fluorophores and short post-excitation durations, the Padé approximant's performance diminishes over extended timeframes and higher fluorophore counts. Nonetheless, its viability, even under demanding circumstances, remains noteworthy.

Our presentation of a reasonable analytical approximation holds considerable novelty, enabling facile prediction of spherical nanoparticle properties amidst energy migration and transfer. The high accuracy consistently verified through Monte Carlo simulations in numerous cases emphasizes its innovative contribution to near-"ideal" solutions. This underscores the substantial potential of leveraging Monte Carlo simulations, not only for simple spherical systems but also for more intricate core–shell nanoparticle configurations marked by higher complexities, where conventional analytical models often yield results that are only approximations.

In particular, our results demonstrate that the Monte Carlo technique can be effectively extended to model three- and four-component fluorescent systems on a nanosphere. This opens up possibilities for achieving a fully tunable system over the visible spectrum. The ability to accurately simulate and predict the behavior of such complex systems holds significant promise for the design and optimization of advanced photonic and optoelectronic devices.

Future studies will delve deeper into these multi-component systems, aiming to refine our understanding of energy transfer dynamics in even more intricate configurations. By continuing to leverage the Monte Carlo approach alongside the three-body Padé approximant, we anticipate uncovering further insights into the interplay of multiple excitations and their interactions in confined spaces. These efforts will pave the way for the development of new materials and technologies that exploit these sophisticated energy transfer mechanisms.

In conclusion, this study makes a significant contribution to the theoretical understanding of excitation transfer and trapping in disordered systems. The novel application of the three-body Padé approximant technique to two-component systems, validated by Monte Carlo simulations, opens up new avenues for research and development in materials science and related fields. Future work will focus on further refining the model, exploring its applications to other types of disordered systems, and extending the theoretical framework to include additional complexities such as varying donor–acceptor interactions and more intricate spatial configurations.

### Supplementary Information

Supplementary Information.

## Data Availability

The datasets used and analysed during the current study available from the corresponding author on reasonable request.
