# Supplementary material for: Theoretical model of donor–donor and donor–acceptor energy transfer on a nanosphere
Source: Sci Rep. 2024 Aug 15;14:18926. doi: 10.1038/s41598-024-69718-4 (PMC11327329; doi:10.1038/s41598-024-69718-4)
Supplement: Supplementary file 1 — Supplementary Information. [file 41598_2024_69718_MOESM1_ESM.docx]

Supplementary material for

**Theoretical model of donor – donor and donor-acceptor energy transfer on a nanosphere**

**Anna Synak^a*^, Leszek Kułak^b^ and Piotr Bojarski^a^**

^*^ corresponding author: [anna.synak@ug.edu.pl](mailto:anna.synak@ug.edu.pl)

^a^ University of Gdańsk, Faculty of Mathematics, Physics and Informatics, Wita Stwosza 57, 80-308 Gdańsk, Poland

^b^ Gdańsk University of Technology, Faculty of Technical Physics and Applied Mathematics, Narutowicza 11/12, 80-952 Gdańsk, Poland.

**DENSITY EXPANSION OF THE GREEN FUNCTION**

**S.1. General equations**

In the system consisting 𝑁 donors and M acceptors located on a sphere with a finite radius $R$ and surface area 𝑆, the Fourier-Laplace transform of the Green’s function $\hat{G}^{DA}\left( t \right)$ can be expressed in terms of the molecules' surface density as

$$\hat{G}^{DA}\left( \boldsymbol{k},\epsilon,N,M \right)=\frac{M}{S}\Lambda_{1}^{DA}\left( \boldsymbol{k},\epsilon,S \right)+\frac{\left( N-1 \right)M}{S^{2}}\Lambda_{2}^{DA}\left( \boldsymbol{k},\epsilon,S \right)+\frac{M\left( M-1 \right)}{S^{2}}\Lambda_{3}^{DA}\left( \boldsymbol{k},\epsilon,S \right)$$

$$+\frac{M\left( M-1 \right)\left( M-2 \right)}{S^{3}}\Lambda_{4}^{DA}\left( \boldsymbol{k},\epsilon,S \right)+\frac{\left( N-1 \right)\left( N-2 \right)M}{S^{3}}\Lambda_{5}^{DA}\left( \boldsymbol{k},\epsilon,S \right)+\ldots(S1)$$

The first term $\Lambda_{1}^{DA}\left( \boldsymbol{k},\epsilon,S \right),$ of the truncated density expansion of Green's function, $\hat{G}^{DA}\left( \boldsymbol{k},\epsilon,N,M \right),$ can be determined by substituting $N=1$, $M=1$ into Eq. (S1), resulting in $\Lambda_{1}^{DA}\left( \boldsymbol{k},\epsilon,S \right)=S \hat{G}^{DA}\left( \boldsymbol{k},\epsilon,N=1,M=1 \right).$ Utilizing the definition of the Green function (refer to Eq.(14)), for $N=1, M=1$ we obtain $\hat{G}^{DA}\left( \boldsymbol{k},N=1,M=1,\epsilon\right){=\left\langle{exp\left( i\boldsymbol{k}\boldsymbol{r}_{12} \right)\left[ \left( \mathbb{E}\boldsymbol{-}\boldsymbol{W} \right)^{-1} \right]}_{21} \right\rangle}_{\mathfrak{R}} ,$where the 2-dimensional matrix $\mathbb{E}\boldsymbol{-}\boldsymbol{W}$ is defined as follows

$$\mathbb{E-}\boldsymbol{W=}\left[ \begin{matrix} \epsilon_{D}+w_{x_{2}x_{1}}^{DA} & 0 \\ \boldsymbol{-}w_{x_{2}x_{1}}^{DA} & \epsilon_{A} \end{matrix} \right]\boldsymbol{,}(S2)$$

where $\epsilon_{D} = \epsilon+\frac{1}{\tau_{0D}}, \epsilon_{A} = \epsilon+\frac{1}{\tau_{0A}} .$

By evaluating the relevant element of the inverse matrix $\left[ \left( \mathbb{E}\boldsymbol{-}\boldsymbol{W} \right)^{-1} \right]_{21}$ in the limit as $\boldsymbol{k}$ approaches $\boldsymbol{0}$, we obtain

$$\Lambda_{1}^{DA}\left( \epsilon\right)=\frac{1}{\epsilon_{A}} \int_{S\in R^{3}} d\boldsymbol{r}_{12}\frac{w_{x_{2}x_{1}}^{DA}}{\epsilon_{D}+w_{x_{2}x_{1}}^{DA}}. \left( S3 \right)$$

The second term, $\Lambda_{2}^{DA}\left( \boldsymbol{k},\epsilon,S \right),$in the truncated density expansion can be obtained by substituting $N=2$, $M=1$into Eq. (S1), yielding $\Lambda_{2}^{DA}\left( \boldsymbol{k},\epsilon,S \right)=S^{2} \hat{G}^{DA}\left( \boldsymbol{k},\epsilon,N=2,M=1 \right)-S \Lambda_{1}^{DA}\left( \boldsymbol{k},\epsilon,S \right).$ In this case, the 3-dimensional matrix $\mathbb{E}\boldsymbol{-}\boldsymbol{W}$ is defined as follows

$$\mathbb{E-}\boldsymbol{W=}\left[ \begin{matrix} \epsilon_{D}+w_{x_{2}x_{1}}^{DD}+w_{x_{3}x_{1}}^{DA} & \boldsymbol{-}w_{x_{1}x_{2}}^{DD} & 0 \\ \boldsymbol{-}w_{x_{2}x_{1}}^{DD} & \epsilon_{D}+w_{x_{1}x_{2}}^{DD}+w_{x_{3}x_{2}}^{DA} & 0 \\ \boldsymbol{-}w_{x_{3}x_{1}}^{DA} & \boldsymbol{-}w_{x_{3}x_{2}}^{DA} & \epsilon_{A} \end{matrix} \right]\boldsymbol{.}(S4)$$

Upon calculating the appropriate element of the inverse matrix $\left[ \left( \mathbb{E}\boldsymbol{-}\boldsymbol{W} \right)^{-1} \right]_{31},$we obtain

$$\Lambda_{2}^{DA}\left( \epsilon\right)=\frac{1}{\epsilon_{A}}\int_{S\in R^{3}} d\boldsymbol{r}_{12}\int_{S\in R^{3}} d\boldsymbol{r}_{13}\left\{ C\left( \boldsymbol{r}_{12},\boldsymbol{r}_{13},\epsilon\right)-\frac{w_{x_{3}x_{1}}^{DA}}{\epsilon_{D}+w_{x_{3}x_{1}}^{DA}} \right\}. (S5)$$

After performing basic algebraic manipulations, the integrand $C\left( \boldsymbol{r}_{12},\boldsymbol{r}_{13},\epsilon\right)$ in Eq. (S5) is given by

$$C\left( \boldsymbol{r}_{12},\boldsymbol{r}_{13},\epsilon\right)=\frac{C_{L}\left( \boldsymbol{r}_{12},\boldsymbol{r}_{13},\epsilon\right)}{C_{M}\left( \boldsymbol{r}_{12},\boldsymbol{r}_{13},\epsilon\right)}, (S6)$$

where

$$C_{L}\left( \boldsymbol{r}_{12},\boldsymbol{r}_{13},\epsilon\right)={\epsilon_{D}w_{x_{3}x_{1}}^{DA}+ w}_{x_{2}x_{1}}^{DD}w_{x_{3}x_{2}}^{DA}+w_{x_{2}x_{1}}^{DD}w_{x_{3}x_{1}}^{DA}+ {w_{x_{3}x_{2}}^{DA} w}_{x_{3}x_{1}}^{DA} (S7)$$

$C_{M}\left( \boldsymbol{r}_{12},\boldsymbol{r}_{13},\epsilon\right)=\left( \epsilon_{D} \right)^{2}+\epsilon_{D}\left( 2w_{x_{2}x_{1}}^{DD}+w_{x_{3}x_{1}}^{DA}+w_{x_{3}x_{2}}^{DA} \right)+w_{x_{2}x_{1}}^{DD}w_{x_{3}x_{2}}^{DA}+w_{x_{3}x_{1}}^{DA}w_{x_{2}x_{1}}^{DD}+w_{x_{3}x_{1}}^{DA}w_{x_{3}x_{2}}^{DA}$(S8)

The third term, $\Lambda_{3}^{DA}\left( \boldsymbol{k},\epsilon,S \right),$in the truncated density expansion can be determined by substituting $N=1$, $M=2$ into Eq. (S1), resulting in $\Lambda_{3}^{DA}\left( \boldsymbol{k},\epsilon,S \right)=\frac{S^{2}}{2} \hat{G}^{DA}\left( \boldsymbol{k},\epsilon,N=1,M=2 \right)-S \Lambda_{1}^{DA}\left( \boldsymbol{k},\epsilon,S \right).$

The matrix $\mathbb{E}\boldsymbol{-}\boldsymbol{W}$ in the definition of the Green function $\hat{G}^{DA}\left( \boldsymbol{k},N=1,M=2,\epsilon\right)$ is given by

$$\mathbb{E-}\boldsymbol{W=}\left[ \begin{matrix} \epsilon_{D}+w_{x_{2}x_{1}}^{DA}+w_{x_{3}x_{1}}^{DA} & 0 & 0 \\ \boldsymbol{-}w_{x_{2}x_{1}}^{DA} & \epsilon_{A}+w_{x_{3}x_{2}}^{AA} & \boldsymbol{-}w_{x_{2}x_{3}}^{AA} \\ \boldsymbol{-}w_{x_{3}x_{1}}^{DA} & \boldsymbol{-}w_{x_{3}x_{2}}^{AA} & \epsilon_{A}+w_{x_{2}x_{3}}^{AA} \end{matrix} \right]\boldsymbol{.}(S9)$$

Upon calculating the appropriate element of the inverse matrix $\left[ \left( \mathbb{E}\boldsymbol{-}\boldsymbol{W} \right)^{-1} \right]_{31}$, we obtain

$$\Lambda_{3}^{DA}\left( \epsilon\right)=\frac{1}{\epsilon_{A}}\int_{S\in R^{3}} d\boldsymbol{r}_{12}\int_{S\in R^{3}} d\boldsymbol{r}_{13}\left\{ D\left( \boldsymbol{r}_{12},\boldsymbol{r}_{13},\epsilon\right)-\frac{w_{x_{3}x_{1}}^{DA}}{\epsilon_{D}+w_{x_{3}x_{1}}^{DA}} \right\}. (S10)$$

where the integrand $D\left( \boldsymbol{r}_{12},\boldsymbol{r}_{13},\epsilon\right)$in Eq.(S10) is defined as follows

$$D\left( \boldsymbol{r}_{12},\boldsymbol{r}_{13},\epsilon\right)=\frac{D_{L}\left( \boldsymbol{r}_{12},\boldsymbol{r}_{13},\epsilon\right)}{D_{M}\left( \boldsymbol{r}_{12},\boldsymbol{r}_{13},\epsilon\right)}, (S11)$$

$$D_{L}\left( \boldsymbol{r}_{12},\boldsymbol{r}_{13},\epsilon\right)={\epsilon_{A}w_{x_{3}x_{1}}^{DA}+ w}_{x_{2}x_{1}}^{DA}w_{x_{3}x_{2}}^{AA}+ {w_{x_{3}x_{2}}^{AA} w}_{x_{3}x_{1}}^{DA}, (S12)$$

$D_{M}\left( \boldsymbol{r}_{12},\boldsymbol{r}_{13},\epsilon\right)=\left( \epsilon_{D}+w_{x_{2}x_{1}}^{DA}+w_{x_{3}x_{1}}^{DA} \right)\left( \epsilon_{A}+2w_{x_{3}x_{2}}^{AA} \right).$(S13)

**S.2. Numerical calculations**

In further discussion, we delve into a scenario where the interaction between the excited donor $D^{*}$ with other donors $D$ and acceptors $A$ follows a dipole-dipole nature, leading to energy migration and non-radiative energy transfer. Under this assumption, the transition probability per unit time for these physical processes is expressed as

$$w_{x_{i}x_{j}}^{DD}=\frac{1}{\tau_{0D}}\left( \frac{R_{0}^{DD}}{r_{ij}} \right)^{6}, w_{x_{i}x_{j}}^{DA}=\frac{1}{\tau_{0D}}\left( \frac{R_{0}^{DA}}{r_{ij}} \right)^{6} . \left( S14 \right)$$

Here, $\tau_{0D}$ denotes the real average lifetime of donor in the excited state when the concentration of molecules tends to zero, and $R_{0}^{DD}$ and $R_{0}^{DA}$ represents the critical radius (distance) for energy migration and transfer, respectively and $r_{ij}$ is the distance between molecules.

Introducing new functions:

$$f_{2}\left( \epsilon\right)=\frac{1}{S} \int_{S\in R^{3}} d\boldsymbol{r}_{12}\frac{w_{x_{2}x_{1}}^{DA}}{\epsilon_{D}+w_{x_{2}x_{1}}^{DA}}, (S15)$$

$$f_{3}\left( \epsilon\right)=\frac{1}{S^{2}}\int_{S\in R^{3}} d\boldsymbol{r}_{12}\int_{S\in R^{3}} d\boldsymbol{r}_{13}\left\{ \epsilon_{A} C\left( \boldsymbol{r}_{12},\boldsymbol{r}_{13},\epsilon\right)-\frac{w_{x_{3}x_{1}}^{DA}}{\epsilon_{D}+w_{x_{3}x_{1}}^{DA}} \right\}, (S16)$$

$$g_{3}\left( \epsilon\right)=\frac{1}{S^{2}}\int_{S\in R^{3}} d\boldsymbol{r}_{12}\int_{S\in R^{3}} d\boldsymbol{r}_{13}\left\{ \epsilon_{A} D\left( \boldsymbol{r}_{12},\boldsymbol{r}_{13},\epsilon\right)-\frac{w_{x_{3}x_{1}}^{DA}}{\epsilon_{D}+w_{x_{3}x_{1}}^{DA}} \right\}, (S17)$$

where $C\left( \boldsymbol{r}_{12},\boldsymbol{r}_{13},\epsilon\right)$and $D\left( \boldsymbol{r}_{12},\boldsymbol{r}_{13},\epsilon\right)$ are defined in Eq.(S6) and Eq.(S11), respectively. Inserting the truncated to the three body terms density expansion of the Green’s function (see Eq. (S1)) into the self-consistent equation Eq.(15), we obtain the following expression for the donor fluorescence decay $\hat{G}^{D}\left( \epsilon\right)$

$$\hat{G}^{D}\left( \epsilon\right)=\frac{1}{\epsilon_{D}}\left( 1-M f_{2}\left( \epsilon\right)-\left( N-1 \right)Mf_{3}\left( \epsilon\right)- M\left( M-1 \right) g_{3}\left( \epsilon\right) \right). (S18)$$

**S.2.1. 2-body approximation**

Substituting the explicit form of the transfer rate $w_{x_{2}x_{1}}^{DA}$into the definition of the function $f_{2}\left( \epsilon\right)$ we have

$$f_{2}\left( \epsilon\right)=\frac{1}{S \epsilon_{D}}\frac{\left( R_{0}^{DA} \right)^{6}}{\tau_{0D}} \int_{S\in R^{3}} d\boldsymbol{r}_{12}\frac{1}{\left( r_{12} \right)^{6}+\frac{1}{\epsilon_{D}\tau_{0D}}\left( R_{0}^{DA} \right)^{6}}. (S19)$$

Considering that on a sphere of radius $R$, $r_{12}=\sqrt{2R^{2}\left( 1-cos\theta\right)}$, where $\theta$ is the angle between the position vectors of the molecules, after substituting $x={sin}^{2}\left( \frac{\theta}{2} \right),$the above integral can be solved exactly to give

$$f_{2}\left( \epsilon\right)=\frac{a}{6} \left\{ ln\left| \frac{\left( 1+a \right)^{2}}{1-a+a^{2}} \right|+2\sqrt{3}\left( arctg\left( \frac{2-a}{a\sqrt{3}} \right)+\frac{\pi}{6} \right) \right\} , (S20)$$

where $a=\frac{1}{4} \left( \frac{R_{0}^{DA}}{R} \right)^{2}\left( \frac{1}{\epsilon_{D} \tau_{0D}} \right)^{1/3}.$

If we measure time in units of $\tau_{0D}$ (i.e. $t \to\frac{t}{\tau_{0D}}$, $\epsilon_{D} \to\epsilon+1$), and exclude the natural decay $exp\left( -\frac{t}{\tau_{0D}} \right)$ the function $f_{2}\left( \epsilon\right)$for small $a$ can be expressed using the series expansion of the logarithm and arctangent. The expression becomes

$$ln\left| \frac{\left( 1+a \right)^{2}}{1-a+a^{2}} \right|+2\sqrt{3}\left( arctg\left( \frac{2-a}{a\sqrt{3}} \right)+\frac{\pi}{6} \right)\approx\frac{4\pi}{3}\sqrt{3} . (S21)$$

Therefore, the following approximate expression for $f_{2}\left( \epsilon\right)$ is

$$f_{2}\left( \epsilon\right)\approx\frac{\sqrt{3} \pi}{18} \left( \frac{R_{0}^{DA}}{R} \right)^{2}\epsilon^{-\frac{1}{3}} . (S22)$$

Finally, the 2-body Padé approximant for the donor fluorescence decay is given by

$$\hat{G}^{D}\left( \epsilon\right)\approx\frac{\frac{1}{\epsilon}}{1+M f_{2}\left( \epsilon\right)+\left( M f_{2}\left( \epsilon\right) \right)^{2}}. \left( S23 \right)$$

**S.2.2. 3-body approximation**

The function $f_{3}\left( \epsilon\right),$ defined in Eq. (S16) in spherical coordinates (see Fig. S1), takes the following form


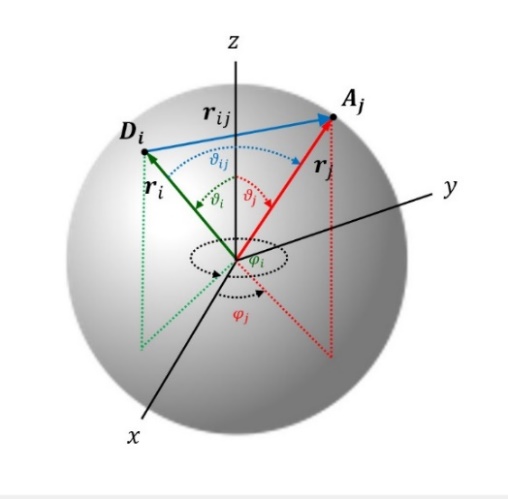


**Figure S1**. Geometry of the investigated system. Vectors $\vec{r}_{i}$, $\vec{r}_{j}$ – define the position of the donor $D_{i}$ and acceptor $A_{j}$ on the nanoparticle surface, the value of vector $\vec{r}_{ij}$– the distance between the donor $D_{i}$and acceptor $A_{j}$. The symbols denoting the angles in the figure correspond to the spherical coordinates of the donor $D_{i}$ and the acceptor $A_{j}$, respectively.

$$f_{3}\left( \epsilon\right)=\frac{1}{16\pi^{2}} \iint_{0}^{\pi} d\vartheta_{1}{d\vartheta}_{2} sin\vartheta_{1}sin\vartheta_{2}\iint_{0}^{2\pi} d\varphi_{1}{d\varphi}_{2}\left\{ C\left( \vartheta_{1},\vartheta_{2},\varphi_{1},\varphi_{2},\epsilon\right)-\frac{w_{x_{3}x_{1}}^{DA}}{\epsilon_{D}+w_{x_{3}x_{1}}^{DA}} \right\}, (S24)$$

where $r_{12}^{2}={2R}^{2}\left( 1-cos\vartheta_{1} \right), r_{13}^{2}={2R}^{2}\left( 1-cos\vartheta_{2} \right)$ and $r_{23}^{2}=2R^{2}\left( 1-cos\vartheta_{1}cos\vartheta_{2}-sin\vartheta_{1}sin\vartheta_{2}cos\left( \varphi_{2}-\varphi_{1} \right) \right).$

Introducing new independent variables, $X=4 \frac{\left( \epsilon_{D} \tau_{0D} \right)^{1/3}}{\xi_{D}^{2}} {sin}^{2}\left( \frac{\vartheta_{1}}{2} \right)$ and $Y=4 \frac{\left( \epsilon_{D} \tau_{0D} \right)^{1/3}}{\xi_{A}^{2}}{sin}^{2}\left( \frac{\vartheta_{2}}{2} \right) ,$ we have

$$f_{3}\left( \epsilon\right)=\frac{1}{64 \pi^{2}} \left( \frac{1}{{\epsilon_{D} \tau}_{0D}} \right)^{2/3}\xi_{A}^{2} \xi_{D}^{2}\int_{0}^{{up}_{1}} dX\int_{0}^{{up}_{2}} dY\iint_{0}^{2\pi} d\varphi_{1}{d\varphi}_{2}\left\{ C\left( X,Y,\varphi_{1},\varphi_{2},\epsilon\right)-\frac{w_{x_{3}x_{1}}^{DD}}{\epsilon_{D}+w_{x_{3}x_{1}}^{DD}} \right\}. (S25)$$

The limits of integration are as follows

$${up}_{1}=4 \frac{\left( \epsilon_{D} \tau_{0D} \right)^{1/3}}{\xi_{D}^{2}}, {up}_{2}=4 \frac{\left( \epsilon_{D} \tau_{0D} \right)^{1/3}}{\xi_{A}^{2}} . (S26)$$

The integral in Eq.(S25) can only be computed numerically. For typical values of the parameter $\epsilon$ in the range used by us with Stehfest’s algorithm for inverting the Laplace transform ($0.1 <\epsilon<2000)$, the value of the integral in the Eq. (S25) is approximately constant within this range (with a relative error of the order of 0.1%). This constant primarily depends on the quotient of the critical radii $R_{0}^{DA}$ and $R_{0}^{DD}$, denoted as

$$\alpha\equiv\frac{\xi_{A}}{\xi_{D}}=\frac{R_{0}^{DA}}{R_{0}^{DD}} .$$

For different values of the parameter $\alpha$, the integral in equation (S25) has been calculated numerically and tabulated and approximated by the following formula

$$f_{3}\left( \epsilon\right)=\frac{1}{64 \pi^{2}} \left( \frac{1}{{\epsilon_{D} \tau}_{0D}} \right)^{2/3}\xi_{A}^{2} \xi_{D}^{2}\mathcal{J}_{3}\left( \alpha, \epsilon\right), (S27)$$

where functions $\mathcal{J}_{3}\left( \alpha, \epsilon\right)$ is defined as

$$\mathcal{J}_{3}\left( \alpha, \epsilon\right)=\frac{1}{\left( a+b \alpha^{2} \right)}, a=0.02712 b=0.04649 (S28)$$

For perfect traps (acceptors), where $R_{0}^{AA}=0,$ the integrand $D\left( \boldsymbol{r}_{12},\boldsymbol{r}_{13},\epsilon\right)$ in the definition of the function $g_{3}\left( \epsilon\right)$ (see Eq.(S17)) simplifies to

$$D\left( \boldsymbol{r}_{12},\boldsymbol{r}_{13},\epsilon\right)=\frac{w_{x_{3}x_{1}}^{DA}}{\epsilon_{D}+w_{x_{2}x_{1}}^{DA}+w_{x_{3}x_{1}}^{DA}} . (S29)$$

In spherical coordinates, for $R_{0}^{AA}=0$, the function $g_{3}\left( \epsilon\right)$ depends only on the angles $\vartheta_{1},\vartheta_{2}$, allowing us to perform integration over the variables $\varphi_{1}$and $\varphi_{2}$ yielding

$$g_{3}\left( \epsilon\right)=\frac{1}{4} \iint_{0}^{\pi} d\vartheta_{1}{d\vartheta}_{2} sin\vartheta_{1}sin\vartheta_{2}\left( D\left( \vartheta_{1},\vartheta_{2},\epsilon\right)-\frac{w_{x_{3}x_{1}}^{DA}}{\epsilon_{D}+w_{x_{3}x_{1}}^{DA}} \right), (S30)$$

where $r_{12}^{2}={2R}^{2}\left( 1-cos\vartheta_{1} \right), r_{13}^{2}={2R}^{2}\left( 1-cos\vartheta_{2} \right).$Using similar substitution as when evaluating the function $f_{3}\left( \epsilon\right),$ we obtain

$$g_{3}\left( \epsilon\right)=\frac{1}{16} \left( \frac{1}{{\epsilon_{D} \tau}_{0D}} \right)^{2/3}\xi_{A}^{4}\iint_{0}^{up} dX dY\left\{ D\left( X,Y,\varphi_{1},\varphi_{2},\epsilon\right)-\frac{w_{x_{3}x_{1}}^{DA}}{\epsilon_{D}+w_{x_{3}x_{1}}^{DA}} \right\}, (S31)$$

where the upper integration limit is equal to u$p=4 \frac{\left( \epsilon_{D} \tau_{0D} \right)^{1/3}}{\xi_{A}^{2}}.$

For typical values of the parameter $\epsilon$ in the range we use in the Stehfest algorithm to invert the Laplace transform ($0.1 <\epsilon<2000$), the value of the integral in Eq. (S31) is approximately constant, with a relative error of 0.1%.

Finally,

$$g_{3}\left( \epsilon\right)=\frac{1}{16} \left( \frac{1}{{\epsilon_{D} \tau}_{0D}} \right)^{2/3}\xi_{A}^{4} \mathcal{K}_{3}, with \mathcal{K}_{3}=-0.82764 . (S32)$$

The approximate expression for the Green function $\hat{G}^{DA}\left( \boldsymbol{k}\boldsymbol{=}\boldsymbol{0},\epsilon\right)$ in the three-body approximation is

$$\hat{G}^{DA}\left( \boldsymbol{k=0},\epsilon\right)=\frac{1}{\epsilon_{A}}\left( M f_{2}\left( \epsilon\right)+\left( N-1 \right)Mf_{3}\left( \epsilon\right)+ M\left( M-1 \right) g_{3}\left( \epsilon\right) \right). (S33)$$

Next, using the self-consistent equation Eq. (15) we obtain

$$\hat{G}^{D}\left( \epsilon\right)=\hat{G}^{SD}\left( \epsilon\right)+\hat{G}^{DD}\left( \boldsymbol{k=0},\epsilon\right)=\frac{1}{\epsilon_{D}}\left( 1-M f_{2}\left( \epsilon\right)-\left( N-1 \right)Mf_{3}\left( \epsilon\right)- M\left( M-1 \right) g_{3}\left( \epsilon\right) \right) . (S34)$$

Finally, the three-body Padé approximant for $\hat{G}^{D}\left( \epsilon\right)$ is

$$\hat{G}^{D}\left( \epsilon\right)=\frac{\frac{1}{\epsilon_{D}}}{1+M f_{2}\left( \epsilon\right)+\left( M f_{2}\left( \epsilon\right) \right)^{2}+\left( N-1 \right)Mf_{3}\left( \epsilon\right)+ M\left( M-1 \right) g_{3}\left( \epsilon\right)} . (S35)$$

**MONTE CARLO METHODS IN NONRADIATIVE EXCITATION ENERGY TRANSPORT**

Monte Carlo simulation is an important research tool in physics. It bridges the gap between theory, analytical description and experiment ^R1-R3, R5-R7^. This technique makes it possible to calculate the values of those quantities characterizing complex physical systems that are difficult to obtain from experiments. In the study of the excitation energy transport phenomena, there is a range of such important quantities that can be calculated using Monte Carlo simulations, which are not possible to be measured directly, for example: the averaged square displacement of excitations during energy migration, the averaged orientation factor.

**A detailed example of Monte Carlo simulation on a spherical nanomolecule**

The basic parameters used in Monte Carlo simulation to study energy transfer in two-component systems are: the number of fluorophores, N donors and M acceptors, critical radii for energy transfer $R_{0}^{DD},R_{0}^{DA},R_{0}^{AA}$, and the radius of the spherical nanoparticle, $R$. Using a pseudo-random number generator with a uniform distribution, molecules are placed randomly on the surface of a sphere with radius $R$. In spherical coordinates, the position vector of $i$-th molecule will be $\boldsymbol{r}_{i}=\left( R, \vartheta_{i},\varphi_{i} \right)$, where $R$is fixed, and the value of the angle $\vartheta_{i}$ is obtained from a uniform distribution of $cos\vartheta_{i}$ in the interval $\left[ -1,1 \right],$ and the value of the angle $\varphi_{i}$ is obtained from a uniform distribution in the interval $\left[ 0, 2\pi\right]$. The donor molecules are labeled 1 through N, and the acceptor molecules N+1 through N+M, respectively.

The method of Monte Carlo simulation used in this work, based on the step-by-step approach^R4^ relies on the application of a pseudo-random number generator to cyclically address two questions

- when, out of a set of predefined processes, a particular event will occur in the simulated system,
- what type of event it will be?

To determine which molecule was initially excited, the photoselection method is commonly used. In our case, for the averaged orientation factor, the initially excited donor molecule is chosen randomly.

In Monte Carlo simulation, the deactivation of the excitation energy in the $j-th$ molecule occurs through one of the following processes

*For donors*

$P_{1}:$ $D^{*}\to D$, nonradiative deactivation of the excited state of the donor with a rate constant of $1/{\tau_{0D}}$ or photon emission,

$P_{2}:$ $D^{*}+D\to D+D^{*}$, energy migration within the donor ensemble with a rate constant of $w_{x_{i}x_{j}}^{DD}$ ,

$P_{3}:$ $D^{*}+A\to D+A^{*}$, energy transfer from the donor to the acceptor with a rate constant of $w_{x_{i}x_{j}}^{DA}.$

*For acceptors*

$P_{1}^{'}:$ $A^{*}\to A$, nonradiative deactivation of the excited state of the acceptor with a rate constant of $1/{\tau_{0A}}$ or photon emission,

$P_{2}^{'}:$ $A^{*}+A\to A+A^{*}$*,* energy migration within the acceptor ensemble with a rate constant of $w_{x_{i}x_{j}}^{AA}$ ,

The rate constants in the above algorithm are as follows

$$w_{x_{i}x_{j}}^{DD}=\frac{1}{\tau_{0D}}\left( \frac{R_{0}^{DD}}{r_{ij}} \right)^{6}, w_{x_{i}x_{j}}^{DA}=\frac{1}{\tau_{0D}}\left( \frac{R_{0}^{DA}}{r_{ij}} \right)^{6}, w_{x_{i}x_{j}}^{AA}=\frac{1}{\tau_{0A}}\left( \frac{R_{0}^{AA}}{r_{ij}} \right)^{6}$$

where $r_{ij}$ is the distance between $i-th$ and $j-th$ molecule.

Next, the total rate constants for the excited $j-th$ donor molecule are calculated

$$c_{1j}=\frac{1}{\tau_{0D}} , c_{2j}=\sum_{i=1, i\neq j}^{N} w_{x_{i}x_{j}}^{DD} , c_{3j}=\sum_{i=N+1}^{N+M} w_{x_{i}x_{j}}^{DA} , {c_{j}=c}_{1j}+c_{2j}+c_{3j}$$

and for the excited $i-th$ acceptor molecule

$$c_{1i}^{'}=\frac{1}{\tau_{0A}} , c_{2i}^{'}=\sum_{j=N+1,i\neq j}^{N+M} w_{x_{j}x_{i}}^{AA} , c_{i}^{'}=c_{1i}^{'}+c_{2i}^{'}$$

The time $t$, at which any of the simulated processes occurs is found by inverting the probability distribution function (CDF), $p_{j}\left( t, P_{k} \right)dt$, of the event that, if the $j-th$ molecule is excited at a given time $t$, the process $P_{k}$will occur within the time interval $\left( t, t+dt \right)$

$$p_{j}\left( t \right)=\sum_{k=1}^{3} p_{j}\left( t, P_{k} \right)=c_{j} exp\left( -c_{j}t \right)$$

Generating a random number $r_{1j}$from the interval $\left[ 0, 1 \right]$ and inverting the cumulative distribution function results in

$$\int_{0}^{t_{j}} p_{j}\left( t \right)dt=r_{1j} , t_{j}=-\left( 1/c_{j} \right) ln\left( 1-r_{1j} \right)$$

In the next step, the process that occurred at time $t_{j}$ is determined. Another random number $r_{2j}$ from the interval $\left[ 0, 1 \right]$is generated and an index value $k$ is sought for which the following inequality is satisfied

$$\sum_{i=1}^{k-1} c_{ij}<r_{2j}c_{j}\leq\sum_{i=1}^{k} c_{ij} , k=1,2,3.$$

Depending on the value of the index $k$ , the corresponding process occurs

- For $k=1$, the excited donor molecule emits a photon or undergoes non-radiative decay to the ground state. This process terminates the current simulation run.
- For $k=2$or $k=3$, an energy migration or transfer process occurs, and it is necessary to determine which molecule is now excited. A random number $r_{3j}$ from the interval $\left[ 0, 1 \right]$ is generated, which is used to find a natural number $n$, that satisfies one of the following inequalities

$$\sum_{i=1}^{n-1} w_{x_{i}x_{j}}^{DD}<r_{3j}c_{2j}\leq\sum_{i=1}^{n} w_{x_{i}x_{j}}^{DD} , for k=2, n\leq N,$$

or

$$\sum_{i=N+1}^{n-1} w_{x_{i}x_{j}}^{DA}<r_{3j}c_{3j}\leq\sum_{i=N+1}^{n} w_{x_{i}x_{j}}^{DA} , for k=3, n>N,$$

where $n$ is the number of the next excited molecule.

If a donor is excited $\left( k=2 \right)$, we calculate the total rate constant $c_{j}$ for the currently excited donor molecule. The next occurrence of the corresponding physical process (deactivating the excitation) is determined, along with its type. When an acceptor molecule is excited $\left( k=3 \right)$ due to energy transfer from a donor, it is necessary to ascertain whether process $P_{1}^{'}$ (photon emission) or $P_{2}^{'}$ (energy migration within the acceptor ensemble) occurs. This determination is made by generating another random number $r_{4j}$ from the interval $\left[ 0, 1 \right]$, and finding the value of the index $k^{'}$ for which the following inequality is satisfied

$$\sum_{l=1}^{k^{'}-1} c_{li}^{'}<r_{4j}c_{i}^{'}\leq\sum_{l=1}^{k^{'}} c_{li}^{'} , k^{'}=1,2.$$

If $k^{'}=1$, the excited acceptor molecule emits a photon or undergoes non-radiative decay to the ground state. This process also terminates the current simulation run.

If $k^{'}=2$, the energy migration process occurs within the acceptor ensemble, and it is necessary to determine which acceptor molecule is now excited. A random number $r_{5j}$ from the interval $\left[ 0, 1 \right]$is generated, which is used to find a natural number $m$ that satisfies the following inequality

$$\sum_{l=N+1}^{m-1} w_{x_{l}x_{i}}^{AA}<r_{5j}c_{2i}^{'}\leq\sum_{l=N+1}^{m} w_{x_{l}x_{i}}^{AA} , m>N,$$

where $m$ is the number of the next excited acceptor molecule. Knowing which acceptor molecule is currently excited, we calculate the total rate constants $c_{i}^{'}$for it and determine the next time $t$, at which the corresponding physical process (deactivating the excitation) will occur and what type of process it will be. This procedure continues until a photon emission process occurs, which terminates the current simulation run.

The donor decay curve is obtained in a way similar to a real experiment. The time scale $T$ (e.g. $[0, T=5]$) is divided into an appropriate number of intervals (e.g. $k_{max}=$ $10000$). If photon emission at the time $t_{i}$ is “observed”, then the number of photons is increased in the respective “channel”. Finally, the normalized decay curve (histogram) is obtained using a formula

$$I\left( t_{k} \right)=1-\left( {\sum_{j=1}^{k} n_{j}}/{\sum_{j=1}^{k_{max}} n_{j}} \right), t_{k}=\left( \frac{k}{k_{max}} \right) T, k=1,.., k_{max}$$

where $n_{k}$ denotes the number of photons in the $k-th$ channel, $k_{max}$ is the total number of all channels, $T$ is total observation time.

The final results are obtained by averaging over a sufficiently large number of spatial and angular configurations of the simulated molecules. Finally, the Monte Carlo simulation is considered completed when the changes in the variance of individual observables do not exceed a small value, for example, 0.1%.


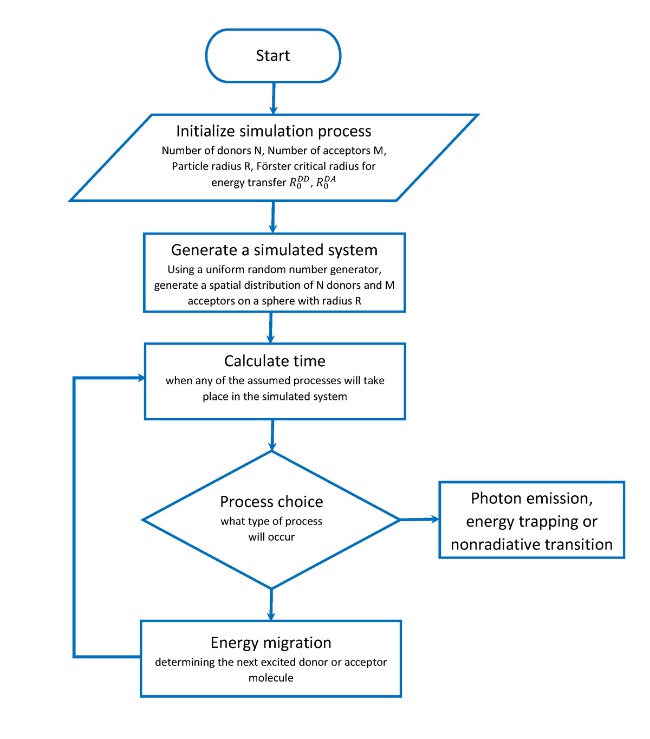


**Figure S2**. Scheme of Monte Carlo simulations.

**References**

[R1] Fishman, G.S. Monte Carlo Concepts, Algorithms and Applications, Springer-Verlag, New York, Inc., (1996).

[R2] Binder, K. Monte Carlo and Molecular Dynamics Simulations in Polymer Science, Oxford University Press, UK (1995).

[R3] Landau, D.P; Binder, K. A Guide to Monte Carlo Simulations in Statistical Physics, Cambridge University Press, UK (2000).

[R4] Gillespie, D.T. Exact stochastic simulation of coupled chemical reactions, *Journal of Physical Chemistry*, **81**, 2340–2361 (1977).

[R5] Bojarski, P.; Kułak, L. Excitation energy transport between the ionic forms of rhodamine B in viscous solutions, *Acta Physica Polonica A* **94**, 5-6, 725-734 (1998).

[R6] Bojarski, P.; Kułak, L.; Walczewska-Szewc, K.; Synak, A.; Marzullo, V. M.; Luini, A. and D’Auria, S. Long-Distance FRET Analysis: A Monte Carlo Simulation Study*, J. Phys. Chem. B*, **115**, 10120–10125 (2011).

[R7] Synak, A.; Grobelna, B.; Kułak, L.; Lewkowicz, A.; and Bojarski, P. Local Dye Concentration and Spectroscopic Properties of Monomer−Aggregate Systems in Hybrid Porous Nanolayers, *J. Phys. Chem. C*, **119**, 14419−14426 (2015).
